# The Impact of Hormonal Contraceptives on Body Weight and Associated Side Effects in Riyadh, Saudi Arabia: A Cross-Sectional Study

**DOI:** 10.7759/cureus.91926

**Published:** 2025-09-09

**Authors:** Hind Khalid Goresh, Rana A Alhebaishi, Fatimah Abdulmohsen Alhejji Alhejji, Samah S Alharbi, Hamoud M Almutairi, Njoud W Albuni, Ftoon Alhomidani

**Affiliations:** 1 Clinical Pharmacy, Buraydah Private Colleges, Buraydah, SAU

**Keywords:** hormonal contraceptive, oral contraceptive, side effects, weight gain, women

## Abstract

Introduction: Public health professionals often use unintended pregnancy as a proxy to understand the community’s need for contraception, highlighting its effectiveness in addressing this issue. However, discontinuation rates and transitions between different contraceptive methods are high, largely due to the emergence of side effects.

Objective: The objective of this study is to assess the association between hormonal contraceptive use and weight gain, as well as other side effects, and evaluate patient satisfaction across different contraceptive methods.

Methods: A cross-sectional observational study was conducted among adult women (≥18 years) attending gynecology clinics in Riyadh in 2023. Women with uncontrolled chronic disease (e.g., diabetes, uncontrolled hypothyroidism, severe polycystic ovary syndrome) were excluded, whereas stable, well-controlled cases under treatment were included. Data were collected using medical records and a structured questionnaire adapted from prior research and piloted locally. Weight change was determined using recorded weights where available, with self-report used in some cases.

Results: Of 500 participants, 324 were eligible for analysis. Combined oral contraceptive (COC) users reported significantly higher mean weight gain compared to users of other hormonal methods. Mood changes and weight gain were the most frequent side effects, while satisfaction was highest among vaginal ring and patch users.

Conclusions: COC users reported greater weight gain and mood-related side effects compared with users of other methods. Satisfaction varied by method, highlighting the importance of individualized counseling to address patient concerns and guide contraceptive choice.

## Introduction

Unplanned pregnancy has garnered significant attention from public health professionals and social scientists. Public health experts often use unintended pregnancies as a proxy to assess the population’s need for contraception, highlighting contraception as an effective solution to this issue. There are two main types of contraceptives available to prevent pregnancy: non-hormonal and hormonal. Non-hormonal methods, such as condoms, the copper intrauterine device (Cu-IUD), and diaphragms, create a barrier between sperm and the egg. Hormonal contraceptives, which include options like oral pills, injections, implants, the levonorgestrel-releasing intrauterine system (LNG-IUS), and vaginal rings, contain estrogen and progesterone or progesterone only [1-3]. These hormonal methods prevent fertilization by thickening cervical mucus to block sperm from reaching an egg or by stopping the release of eggs from the ovaries. They are considered highly effective because they do not rely on patient adherence [4]. The rate of discontinuation and switching between contraception methods is high due to various side effects. A notable side effect is weight gain, which can occur due to fluid retention, increased muscle mass, or fat deposition. In some cases, combined contraceptives may also increase appetite [5]. A national epidemiological health survey found that the prevalence of overweight and obesity in Saudi Arabia was 36. 9% (n = 1,845) and 35.5% (n = 1,775), respectively, with women experiencing higher rates of obesity than men. Being overweight or obese is associated with multiple medical issues [6]. Numerous clinical studies have investigated the relationship between weight gain in different types of hormonal contraceptives and that in non-hormonal ones, concluding that users of hormonal contraceptives experience substantial weight gain. Other side effects associated with oral contraceptives include an increased risk of depression and venous thromboembolism [5,7,8,9]. Concerns related to implants and IUDs include reduced platelet concentration and menstrual irregularities. Additionally, there is a noted increase in headache episodes and changes in lipid profiles among women using oral contraceptives [10,11]. Studies conducted in Saudi Arabia indicate that 51.0% (n = 260) of participants reported weight gain as a side effect, demonstrating a significant association between weight gain and the use of oral contraceptives. Other reported side effects include headaches (n = 138, 27.1%), depression (n = 84, 16.5%), and bleeding (n = 79, 15.6%) [12]. Sergison et al. conducted a meta-analysis of 10 studies involving 2,081 participants using the 52 mg LNG-IUS, including Mirena, to assess the prevalence of side effects. They found the pooled prevalence of headache to be 17.3% (95% CI, 13.8%-21.5%) and the pooled prevalence of weight gain to be 11.6% (95% CI, 8.3%-16.0%) [13]. In the United States, approximately 23.0% (n = 230) of women using hormonal contraception were diagnosed with depression, and about 40.0% (n = 400) experienced irregular bleeding [14].

## Materials and methods

Study design and participants

This was a cross-sectional observational study. Eligible participants were adult women aged 18 years or older using hormonal contraceptives. Women with uncontrolled chronic or metabolic conditions known to affect body weight (e.g., uncontrolled diabetes, severe polycystic ovary syndrome (PCOS), uncontrolled hypothyroidism, renal failure, or chronic corticosteroid use) were excluded. However, women with well-controlled, stable conditions under medical treatment (such as treated hypothyroidism or stable PCOS) were included.

Survey instrument

A researcher-developed questionnaire was used, adapted from items employed in prior contraceptive research [15,16]. Two senior gynecologists reviewed the survey for face validity, and it was piloted among 20 women in Riyadh for clarity and comprehensibility. The final questionnaire included 25 items, grouped into four domains: (i) Socio-demographics (age, education, physical activity, BMI); (ii) Contraceptive history (current and past method use); (iii) Side effects (assessed as binary yes/no); (iv) Satisfaction (measured using a 10-point Likert-type scale).

Operational definitions

Education

This is categorized as no formal education, primary/secondary education, university education, and postgraduate education.

Physical Activity

This is classified as low, moderate, or high based on self-reported frequency and intensity of exercise.

BMI

BMI is categorized using WHO criteria as underweight (<18.5), healthy weight (18.5-24.9), overweight (25-29.9), and obese (≥30). Women with obesity (BMI ≥30) were excluded from the study sample.

Weight measurement

Baseline and follow-up weights were obtained from medical records whenever available. For women without complete records, self-reported weights were used, which we acknowledge as a limitation due to potential recall bias.

Statistical analysis

Continuous variables were presented as mean ± SD and compared using Student’s t-test or regression models. Categorical variables were reported as frequencies and percentages and analyzed using chi-square or Fisher’s exact test as appropriate. A p-value <0.05 was considered statistically significant.

## Results

**Figure 1:**
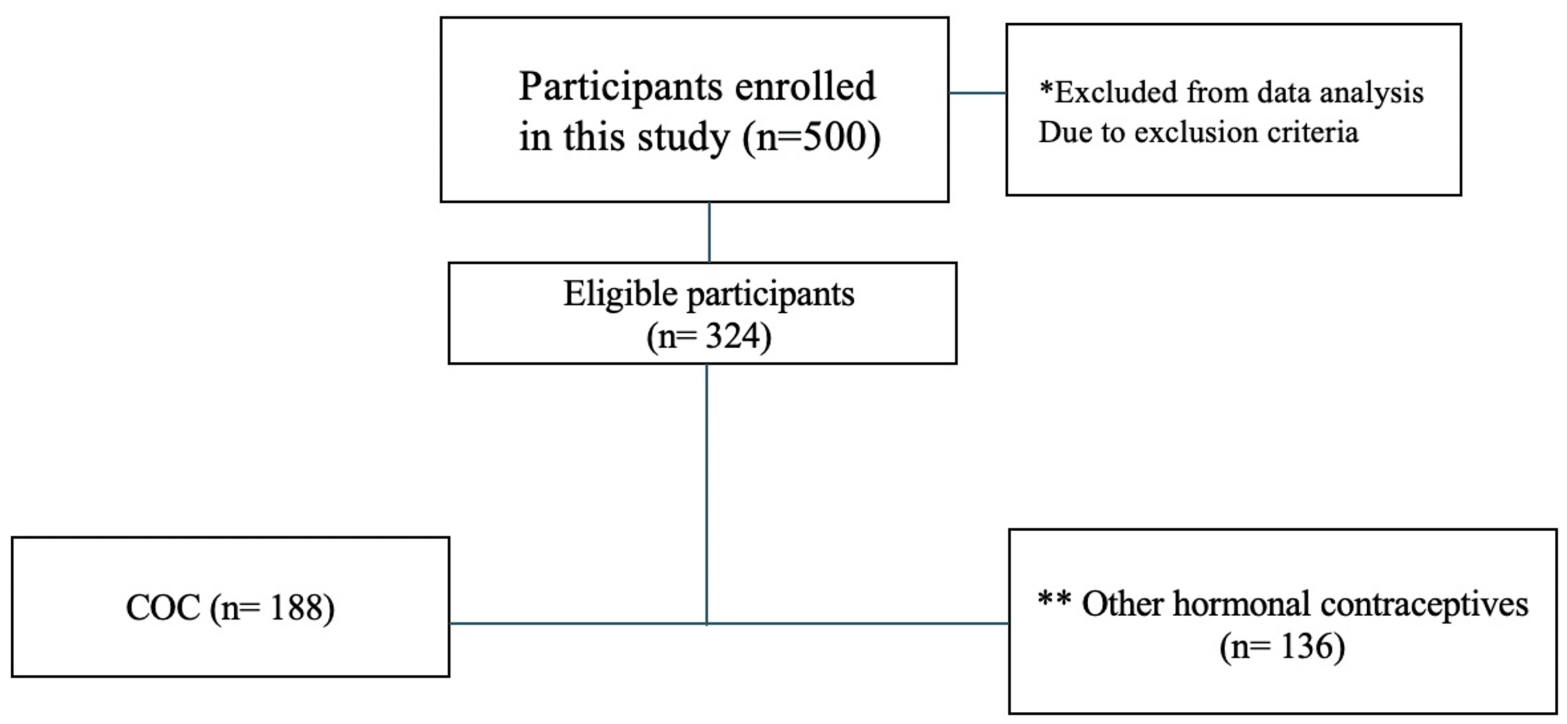
Participant flow diagram showing enrollment, exclusions, and final study population by the contraceptive method. *Chronic disease known to affect weight for example (diabetes, systemic arterial hypertension, hyper/hypothyroidism, renal failure, polycystic ovary syndrome), use of medications known to affect weight like (daily corticosteroids, NSAID), contraindication to estrogen use, and hormonal disturbance; ** Other methods of hormonal contraceptive such as a patch or clinical procedure like implants, intrauterine device, vaginal ring, and injections. COC: Combined oral contraceptive

A total of 500 female participants completed our questionnaire. Of these, 176 (35.2%) were excluded from the data analysis based on the predefined criteria of exclusion. Consequently, 324 (64.8%) participants were eligible and included in the final analysis. Among the eligible participants, there were 188 (58.0%) participants who reported using combined oral contraceptives (COCs), while 136 (42.0%) were using other types of hormonal contraceptives (Figure 1).

Table 1 shows the sociodemographic parameters of the 324 women who were using hormonal contraceptives. Most women were aged 25-34 years (192, 59.3%), which is followed by 35-44 years (85, 26.2%), while only 8.3% (n=27) were aged 18-24. The educational attainment was predominantly academic (235, 72.5%), with a small proportion being illiterate (4, 1.2%). The moderate physical activity was most common (235, 72.5%) among the women. Regarding the BMI, over half of the women were overweight (163, 50.3%), while only 4.3% (n=14) were underweight. COCs were the most used method (188, 58%), which was followed by the IUDs (57, 17.6%) and patches (26, 8%), with method use differing significantly (p = 0.001). Most of the women had no comorbidities (148, 45.7%). PCOS (55, 17.0%) and hypothyroidism (45, 13.9%) were notable comorbidities in other women, which was statistically significant (p = 0.036). Moreover, the satisfaction levels were high in 138 women (42.6%) and moderate in 136 women (42%).

**Table 1:** Socio-demographic characteristics of the women and types of contraceptive methods used (n = 324). *p < 0.05 is considered statistically significant. COC: Combined oral contraceptive; IUD: intrauterine device; SC: subcutaneous; IM: intramuscular

Demographic Category	Variable	N	%	p-value
Age	18–24 years	27	8.3	0.3
	25–34 years	192	59.3	
	35–44 years	85	26.2	
	45–54 years	20	6.2	
Education level	Illiterate	4	1.2	0.45
	School	43	13.3	
	Academic	235	72.5	
	Postgraduate	42	13.0	
Physical activity	Low	38	11.7	0.25
	Moderate	235	72.5	
	High	51	15.7	
BMI	Thin (<18.5)	14	4.3	0.63
	Normal (18.6–24.9)	147	45.4	
	Overweight (25–29.9)	163	50.3	
Types of contraceptives	COC	188	58.0	0.001*
	Progesterone pills	22	6.8	
	IM injections	14	4.3	
	SC implants	13	4.0	
	Vaginal ring	4	1.2	
	IUD	57	17.6	
	Patches	26	8.0	
Comorbidity	None	148	45.7	0.036*
	Hypertension	27	8.3	
	Diabetes	31	9.6	
	Hyperthyroidism	18	5.6	
	Hypothyroidism	45	13.9	
	Polycystic ovary	55	17.0	
Satisfaction with contraceptives	None	23	7.1	0.52
	Low	27	8.3	
	Moderate	136	42.0	
	High	138	42.6	

Body weight change by the contraceptive method

Table 2 shows the comparison of weight gain between the women who were using the COC and other hormonal contraceptive methods. Notably, among 188 women who were using the COC method, 66.5% (n=125) reported that there was a mean weight gain of 5 kg (SD = 0.44). Among 136 women who were using other hormonal methods, 47.1% (n=64) reported that there was a weight gain of 3.3 kg (SD = 0.42). There was a significant increase in weight among women using COC compared to the women who were using other types of contraceptives (p = 0.001).

**Table 2:** Weight increases in COC vs other types of contraceptives. *p < 0.05 is considered statistically significant. COC: Combined oral contraceptive

Method	N	Confirmed Weight Increase N (%)	Mean Weight Gain (kg)	SD	Test	p-value
COC	188	125 (66.5%)	5	0.44	2.7	0.001*
Others	136	64 (47.1%)	3.3	0.42		

Perceived side effects of hormonal contraceptives among the users

**Figure 2:**
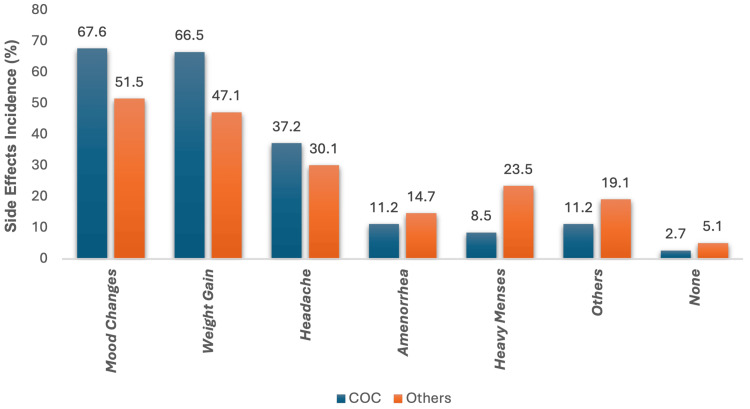
Bar chart showing a comparison of the incidence of side effects between COC (n=188) and other contraceptive types (n=136). COC: Combined oral contraceptive

Figure 2 compares the side effects between the users of COC and other hormonal contraceptives. The mood changes were the most frequently reported side effect with both methods, as 67.6% (n=127) of COC users and 51.5% (n=70) of other contraceptive users reported mood changes. The second most common side effect was weight gain, which was experienced by 66.5% (n = 125) of COC users as compared to 47.1% (n = 64) of the other group. Moreover, headache was reported by 37.2% (n = 70) among COC users and 30.1% (n = 41) who used other hormonal methods. Furthermore, heavy menstrual bleeding was more prevalent in the other hormonal methods group with 23.5% (n = 32), whereas only 8.5% (n = 16) of COC users had reported this issue. Amenorrhea was observed in 11.2% (n = 21) of COC users as compared to 14.7% (n = 20) among others. Notably, other adverse effects were shown by 11.2% (n = 21) in the COC group as compared to 19.1% (n = 26) of the other contraceptives group. There are only 2.7% (n = 5) of women among COC who reported no adverse effects; similarly, 5.1% (n = 7) of the other hormonal contraceptives group reported no side effects at all.

Participant stratification of hormonal contraceptive users

**Figure 3:**
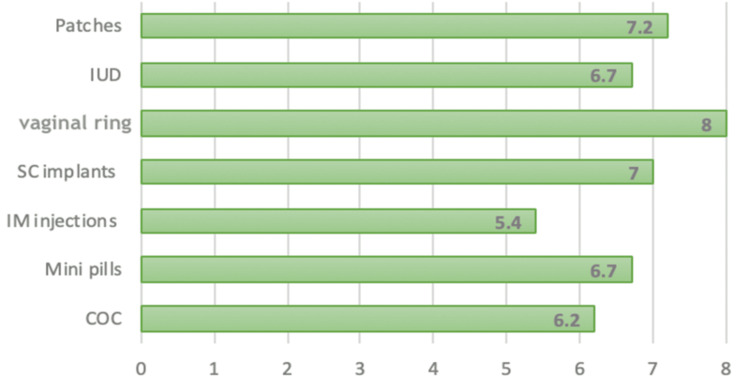
Line graph showing the satisfaction score of patients using different types of contraceptives.

Figure 3 shows the mean satisfaction scores of the patients who were using the various types of contraceptive methods. The highest satisfaction was reported by the users of the vaginal ring, with a score of 8.0. It is followed by the users of the contraceptive patches (7.2) and subcutaneous (SC) implants (7.0). Intrauterine devices (IUDs) and mini pills both had the same mean satisfaction score of 6.7. The COC users reported a slightly lower satisfaction score of 6.2, while intramuscular (IM) injection users showed the lowest satisfaction level at 5.4.

## Discussion

In recent years, the prevalence of hormonal contraceptive use has shown a rapid increase due to their greater effectiveness and appropriateness across the reproductive life cycle, where women seek to choose this method to control pregnancy. These contraception types include pills, intrauterine devices, injectables, implants, and condoms. Each method has its impact on physical and psychological health. Therefore, women often attempt to change the contraceptive type [17,18].

Our study demonstrates that there was a significantly higher body weight increase in users of combined oral contraceptives, with an average gain of 5.0 kg, compared to other types. This finding corresponds with previous research that found combined contraceptive pill users experienced weight increases of 3.98 kg [7], which is considered high compared to other types, while some studies did not find any bodyweight gain compared to placebo or non-user control groups [19,20]. However, we found that the highest percentage of women using hormonal contraceptives was in the age group above 20 years, which is notable as women in this age group tend to be concerned about their weight.

Most of the women experienced the usual side effects in both groups, mainly mood changes, weight gain, headache, heavy menses, and amenorrhea. Other side effects include nausea, leg pain, polycystic ovaries, and hair loss. Mood change was the most frequently experienced side effect in the COC group and the other hormonal contraceptive group, reported by 67.4% (n = 127) and 51.5% (n = 70), respectively [21-23]. A study by Johnson concluded that contraceptive pills could cause mood changes more than other types [24].

Also, a cross-sectional study of 500 participants reported mood changes with oral contraceptive pill use among Iranian women, which appeared consistent with other studies [24,25]. Overall, possible mood-related hormonal contraceptive side effects should be carefully weighed against the profound benefit of hormonal contraceptive methods for safe family planning. Likewise, headache is a typical side effect among OC users; previous studies concluded that 50% of OC users reported headaches [25], which supports our finding that 37.2% (n = 70) of participants experienced headaches as a side effect.

The present study has certain advantages compared to previously published reports from Saudi Arabia [26]. For example, the questions asked in this survey were different, included all types of hormonal contraceptives, and compared the incidence of weight gain and possible side effects.

Study limitations

This study has several limitations that should be acknowledged. Its cross-sectional design prevents establishing causal relationships between contraceptive methods and outcomes. Data were collected from a single tertiary center in Riyadh, which limits generalizability. Women with BMI ≥30 kg/m², chronic conditions, or medications affecting weight were excluded, potentially reducing applicability to broader populations. Self-reported side effects and baseline weight may be prone to recall bias. Unequal group sizes and unmeasured variables, such as duration and specific formulation of contraceptives, may also confound the findings. 

## Conclusions

In this cross-sectional study of women using hormonal contraception in Riyadh, COC users reported greater weight gain and mood-related side effects compared with users of other hormonal methods, while satisfaction was highest among vaginal ring and patch users. These findings emphasize the importance of individualized counseling that addresses potential weight and mood effects and highlights alternative methods for women with specific concerns. Broader, longitudinal studies are required to strengthen the evidence base and guide clinical and policy recommendations in contraceptive care.

## Appendices

**Table 3:** Survey on the effect of hormonal contraceptives on body weight gain and other side effects.

Category	Item	Options/Input
File Info	File Number	____________________
Age (years)	☐ 18–24 ☐ 25–34 ☐ 35–44 ☐ 45–54 ☐ 55+	
Weight (kg)	____________________	
Height (cm)	____________________	
BMI	☐ Underweight <18.5 ☐ Normal 18–24.9 ☐ Overweight 25–29.9	
Weight Change	Did you experience any weight change while using contraceptive?	☐ Yes ☐ No
If Yes, how much (kg)?	____________________	
Education	Education Level	☐ Illiterate ☐ School ☐ Academic ☐ Postgraduate
Physical Activity	Physical Activity Level	☐ Low ☐ Moderate ☐ High
Co-morbid Conditions	Any of the following? (check all that apply)	☐ Hypertension ☐ DM ☐ Dyslipidemia ☐ Hyperthyroidism ☐ Hypothyroidism
☐ PCOS ☐ Thromboembolic disorders ☐ Breast cancer or estrogen-dependent neoplasia		
☐ Known/suspected pregnancy ☐ Benign/malignant liver tumor ☐ Other: ______________		
Medications	List your current medications	1. ____________ 2. ____________ 3. ____________
4. ____________ 5. ____________ 6. ____________		
Contraceptive Use	Ever used any contraceptive method?	☐ Yes ☐ No
If Yes, What Type?	☐ Combined oral contraceptive ☐ Progestin-only pills ☐ Injectable	
☐ Implants ☐ Vaginal ring ☐ IUD ☐ Contraceptive patch		
If No, Why?	☐ Fear of side effects ☐ Cost ☐ Less effective ☐ Forget to use ☐ Other: ___________	
Previously Used Type	☐ Combined oral contraceptive ☐ Progestin-only pills ☐ Injectable	
☐ Implants ☐ Vaginal ring ☐ IUD ☐ Contraceptive patch		
If Stopped, Why?	☐ Headaches ☐ Weight gain ☐ Mood changes ☐ Heavy bleeding ☐ Amenorrhea	
☐ Other: ___________________		
Future Use	Would you consider using contraception in the future if you experience side effects?	☐ Yes ☐ No
If Yes, which method?	____________________	
Pregnancy History	How many times have you been pregnant?	Number: ____________
Satisfaction	Satisfaction with current method (scale 1–10)	____________
Why?	☐ Convenience ☐ Fewer side effects ☐ Easy to use ☐ Reliable ☐ Other: ______________	
Follow-Up	Are you on regular follow-up?	☐ Yes ☐ No
If Yes, how often?	☐ Every 3 months ☐ Every 6 months ☐ Every 12 months	
